# Cognitive processing of sexual cues in asexual individuals and heterosexual women with desire/arousal difficulties

**DOI:** 10.1371/journal.pone.0251074

**Published:** 2021-05-12

**Authors:** Natalie B. Brown, Diana Peragine, Doug P. VanderLaan, Alan Kingstone, Lori A. Brotto

**Affiliations:** 1 Department of Psychology, University of British Columbia, Vancouver, Canada; 2 Department of Psychology, University of Toronto Mississauga, Mississauga, Canada; 3 Child and Youth Psychiatry, Centre for Addiction and Mental Health, Toronto, Canada; 4 Department of Obstetrics and Gynaecology, University of British Columbia, Vancouver, Canada; Ruhr-Universität Bochum, GERMANY

## Abstract

Asexuality is defined as a unique sexual orientation characterized by a lack of sexual attraction to others. This has been challenged, with some experts positing that it is better explained as a sexual dysfunction. Sexual Interest/Arousal Disorder (SIAD) is characterized by absent/reduced sexual interest/arousal paired with personal distress, with two subtypes: acquired and lifelong. Research suggests that while asexuality and acquired SIAD are distinct entities, there may be overlap between asexuality and lifelong SIAD. Findings from studies using eye-tracking and implicit association tasks suggest that these methodologies might differentiate these groups on the basis of their neural mechanisms. However, no study has compared their cognitive processing of sexual cues, and the literature on lifelong SIAD is minimal. The current study tested differences in the cognitive processing of sexual cues between asexual individuals and women with SIAD (lifelong and acquired). Forty-two asexual individuals and 25 heterosexual women with SIAD (16: acquired; 9: lifelong) completed three study components: a visual attention task, a Single Category-Implicit Association Task, and the sex semantic differential. ANOVAs examined group differences in: 1) visual attention to erotic cues, 2) implicit appraisals of sexual words, and 3) explicit appraisals of sex. Women with SIAD displayed a controlled attention preference for erotic images and areas of sexual contact, with longer dwell times to these areas relative to asexual individuals, who did not gaze preferentially at erotic cues. For implicit appraisals, all groups demonstrated negative—neutral implicit associations with sexual words. For explicit appraisals, women with acquired SIAD reported more positive evaluations of sex relative to asexual individuals and women with lifelong SIAD. This project sheds light on key differences between asexuality and low desire, and has implications for best clinical practice guidelines for the assessment of lifelong SIAD.

## Introduction

### Asexuality

Human asexuality is generally defined as a lack of sexual attraction to others [1]. There has been a surge of academic interest in asexuality in response to national probability studies suggesting approximately 0.4–1% of the population identifies as asexual [2–4]. Despite this wave of research, there has not been a corresponding shift in societal acceptance of those who do not experience sexual attraction, with empirical evidence suggesting that asexual individuals experience stigmatization [5, 6]. For instance, college students rated asexual people as less likely to possess traits (e.g., friendliness) or to experience emotions (e.g., affection) than their allosexual (non-asexual) counterparts [6]. Further, asexual people describe pathologization when they disclose their sexual identity to others, often being told they are “unnatural” [5, 7]. The Asexuality Visibility and Education Network (AVEN) aims to reduce the marginalization experienced by this group by facilitating open communication about asexuality, which they conceptualize as a unique sexual orientation [8]. However, scholars have argued that the line between asexuality and sexual dysfunction is not clearly defined [5]. Specifically, the psychological community struggles with the distinction between asexuality and Sexual Interest/Arousal Disorder (SIAD), given both groups’ disinterest in sexual activity [9, 10].

### Asexuality spectrum

The umbrella term “ace” is used to encompass the diversity of experiences of attraction, relationships, and arousal within the asexual community [11–13]. On one end of the ace continuum are individuals who do not report ever experiencing attraction to others, referred to simply as asexual. Gray-As, who experience sexual attraction infrequently or only with certain people or in specific situations, can also be found on this spectrum [11]. There is evidence of substantial gender diversity, as research suggests that a large number of asexual people do not fit the gender binary [14, 15]. For example, a study of 1,268 asexual participants revealed that 16.2% listed a gender identity other than man/woman [15]. Moreover, the 2016 Asexual Community Census indicated that only 74% of 9,331 ace participants identified as “woman/female” or “man/male” [16]. Notably, there is a sex bias within the ace community, as the majority of 2016 Asexual Community Census respondents (i.e., 89%) disclosed being female at birth [16]. However, 33% of ace census respondents reported a gender identity that was not congruent with their sex at birth [16], suggesting higher rates of trans experience relative to the general population.

### Sexual Interest/Arousal Disorder (SIAD)

SIAD is a sexual dysfunction characterized by absent or reduced sexual interest and/or arousal paired with significant personal distress, and is included in the *Diagnostic and Statistical Manual of Mental Disorders* (DSM-5) [17]. The results of a recent nationally representative study of German residents between the ages of 18–75 revealed that approximately 6% of women reported low sexual desire in the past 12 months, which they associated with severe distress [18]. To meet diagnostic criteria for SIAD, women must report an absence or reduction in at least 3 of the following domains for 6 months or longer: interest in sex, sexual thoughts/fantasies, responsive sexual desire, initiation of or receptivity to sexual activity, sexual pleasure, and physical sexual sensations [17]. There are two subtypes of SIAD based on onset: acquired and lifelong. Women with acquired SIAD report having previously experienced satisfactory levels of sexual desire/arousal, followed by a distressing reduction in desire [17]. Conversely, women with lifelong SIAD report always having experienced distressing sexual concerns. The majority of research on low desire focuses on women with acquired rather than lifelong SIAD, resulting in a paucity of data on the latter group and what characteristics differentiate SIAD subtypes.

### Comparing asexuality and SIAD

Given that asexual individuals and women with SIAD both report a lack of sexual desire (and often reduced sexual activity) [19], they are often conflated by the general public and by clinicians treating sexual and/or relationship concerns. However, scholars have challenged this interpretation [19–22]. Importantly, asexual people do not experience their lack of sexual interest as distressing, a hallmark of sexual desire disorders [14, 19, 23]. Further, women with SIAD seek treatment for their sexual concerns, whereas the majority of asexual persons view their orientation as a non-pathological, alternative way of being, and have no wish to be “fixed” [5, 14, 24]. To illustrate, asexual advocates created an AVEN *DSM* Task Force to advise the DSM-5 working group that formulated definitions of sexual dysfunctions. Their report sought to inform diagnostic criteria for sexual desire disorders without contributing to stigma surrounding asexuality, and made two recommendations [24]. First, they asked “attraction to neither males nor females” be added as a sexual orientation. Second, they requested a clause for any desire disorder, specifying that the diagnosis applies only to those who do not identify as asexual.

Another key difference is the onset and duration of disinterest in sex. Qualitative research reveals that many asexual people report “always feeling this way”, whereas those with acquired low desire describe a period of satisfying sexual desire before the onset of low or absent sexual interest [14]. On the other hand, asexual persons and those with lifelong low desire both describe a more longstanding pattern to their absent sexual interests.

Only one study has compared asexual persons and women with a sexual desire disorder on sex-related variables [19], representing a gap in the literature. Notably, asexual individuals reported fewer sexual partners and fantasies, less frequent sexual activity, lower desire and sex-related distress, and fewer depressive symptoms than women with low desire [19]. A follow-up analysis of women with lifelong SIAD revealed that while this group reported more sexual behaviors and higher desire than asexual persons, these differences were smaller than those observed between asexual individuals and women with acquired SIAD. When comparing subtypes, women with lifelong SIAD reported less frequent sexual activity and lower desire than those with acquired SIAD, but disclosed similar sex-related distress [19]. These findings suggest a possible overlap between asexual individuals and women with lifelong sexual concerns.

### Cognitive processing of sexual cues

**Fig 1:**
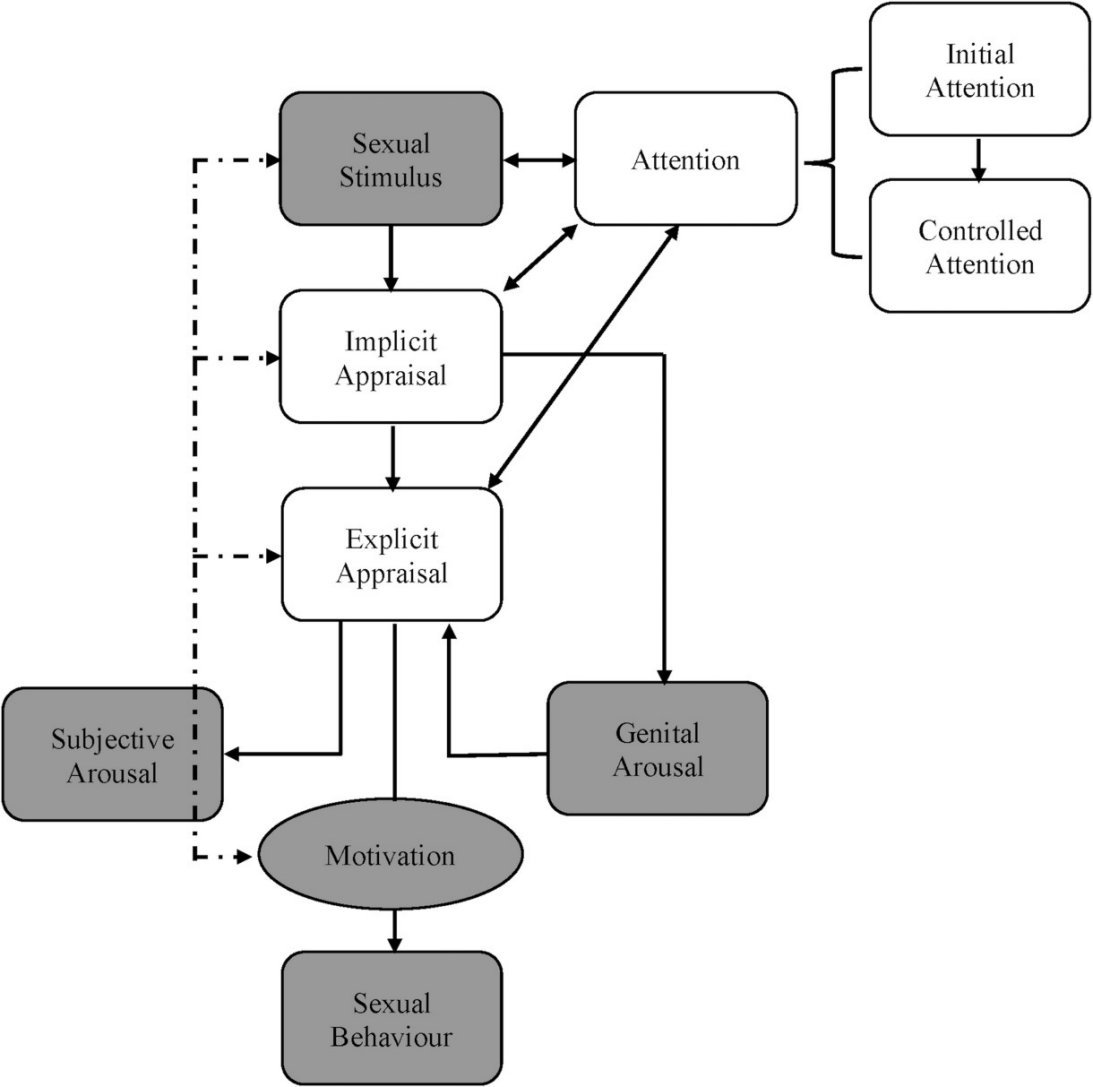
Cognitive-motivational model of sexual response. Adapted from DeWitte [25] and Janssen and colleagues [26].

According to Dewitte’s cognitive-motivational model of sexual response (Fig 1), visual attention to and appraisal of erotic cues are prerequisites for sexual arousal [25]. Visual attention towards sexual stimuli initiates and sustains the sexual response. Controlled attention involves the explicit processing of a cue’s meaning and awareness of sexual arousal [26], and can be quantified by the total time (i.e., dwell time) or number of times (i.e., number of fixations) an individual looks at an item [27]. Gaze behavior acts as a sensitive index of visual attention, and eye-tracking systems continuously assess attentional allocation executed via eye movements, operationalized by fixation data [28]. Research suggests that sexual orientation influences controlled attention, such that individuals gaze for longer periods of time and more often at stimuli depicting their sexually preferred rather than nonpreferred gender [27, 29]. For example, studies employing forced attention paradigms–the simultaneous presentation of two distinct images that differ on variable of interest–revealed that heterosexual women exhibited longer dwell times and made more fixations to images of nude men (i.e., preferred gender) relative to nude women (i.e., nonpreferred gender), whereas lesbian women and heterosexual men showed the opposite pattern [27, 29]. Also, these authors found that participants’ gaze times were positively correlated (*r* = .47-.76) with their self-reported attraction to these images [27]. These findings provide support for the use of controlled attention as an indicator of one’s preferred sexual targets (e.g., men, women). Following this logic, since asexual persons do not experience sexual attraction to others, we predicted that asexual individuals would not gaze preferentially at an image depicting a sexual interaction between actors relative to a non-sexual image.

In contrast, researchers found that heterosexual women with no sexual concerns and women with low desire demonstrated a controlled attention bias for erotic cues [30]. Specifically, when participants viewed images of nude men and women engaging in sexual activity, both the control and low desire samples looked for longer and more often at the actors’ bodies than the background, with no group differences in gaze behavior to these areas [30]. Based on these results, we predicted that heterosexual women with SIAD would have a controlled attention preference for sexual images, exemplified by longer dwell times and a greater number of fixations to erotic relative to neutral scenes. As both lifelong and acquired SIAD impact women who retain their sexual attractions, we predicted no differences in gaze behavior between these groups.

Appraisal (i.e., subjective evaluation) of an erotic cue is another prerequisite of sexual responding identified by Dewitte’s model, incorporating implicit and explicit processes [25]. Implicit appraisals give stimuli emotional meaning, involve encoding and matching stimuli in memory, are not part of conscious awareness, and are based on past experience [25, 26, 31]. Given that asexual persons reported less frequent engagement in sexual activities compared to an allosexual sample with acquired low desire [19], it is logical that sexual cues hold less positive meaning for asexual individuals than heterosexual women with acquired SIAD [14]. In contrast, because asexual individuals and women with lifelong SIAD had comparable low levels of sexual activity, it is possible that these groups share similar implicit appraisals of erotic cues.

The Implicit Association Task (IAT) [32] is the most widely used measure of implicit appraisals, relying on reaction times within a dual classification task to assess the strength of associations held in memory [31]. Researchers have used the IAT to distinguish individuals with different sexual orientations [33–35]. One limitation of the IAT is its measurement of attitudes towards related but separate concepts (e.g., male and female) making it difficult to measure appraisals of concepts with no logical alternative category (e.g., sex). The SC-IAT circumvents this issue by capturing negative and/or positive associations of a single concept [36, 37]. In the first study examining asexual persons’ implicit appraisals, asexual and heterosexual men and women completed a sex SC-IAT [35]. The asexual sample performed the word classification task more efficiently when the words “bad” and “sex” were paired, whereas the heterosexual participants were quicker when “good” and “sex” were paired. Thus, those who identified as asexual had less positive implicit appraisals of erotic cues than the allosexual comparison group.

One study to date used an IAT to examine implicit appraisals of sexual cues in women with clinically low desire relative to heterosexual controls, and found that women with acquired low desire showed less positive associations with sexual imagery [31]. However, this difference was smaller than that observed for asexual and heterosexual persons without sexual concerns [35]. Considering asexual individuals’ lower levels of sexual activity and the aforementioned IAT findings, we expected that asexual participants would have less positive implicit appraisals of sexual cues compared to heterosexual women with acquired SIAD. Notably, women with lifelong SIAD have not been included in IAT studies. Considering evidence that this group reported levels of sexual (in)activity and desire similar to asexual persons [19], we predicted the implicit appraisals of sexual cues would be similar between women with lifelong SIAD and asexual individuals, and both groups having less positive appraisals than women with acquired SIAD.

According to Dewitte’s model [25], from a temporal perspective implicit appraisals of a sexual cue are followed by explicit appraisals (i.e., deliberate evaluations of stimuli) referred to as attitudes. While asexual individuals report a range of attitudes towards sex, from neutral to finding sex aversive, their evaluations of erotic cues are more negative overall than those of allosexual participants [12, 35, 38]. For example, when asexual persons and allosexual controls completed a measure of explicit attitudes (the sex semantic differential) and were asked to place “sex” between 11 pairs of opposing words (e.g., Good/Bad), the asexual group consistently situated sex closer to the negative words than the allosexual group did [35]. For women with sexual concerns, researchers examined discrepancies between the explicit appraisals of erotic cues for heterosexual controls and women with acquired low desire, with mixed findings [31]. In response to one set of erotic images, women with acquired low desire reported more disgust and less desire than the control women, but there were no differences observed for a separate, albeit similar, set of images [31]. Given these results, we expected that asexual individuals would report less positive explicit appraisals of sexual stimuli than heterosexual women with acquired SIAD. Unfortunately, there are currently no published studies examining attitudes towards sex held by women with lifelong low desire, representing a gap in the literature. However, data suggest that self-reported attitudes towards sex are influenced by societal context [39]. Given that women with lifelong and acquired SIAD are embedded in Western culture, which celebrates compulsory sexuality, the widespread assumption that all people are sexual and that sexual relationships are superior to platonic forms of intimacy [40], we predicted that explicit appraisals of sexual cues would be similar in the two SIAD groups.

### Current study

The current study aimed to test differences between samples of individuals who identified as asexual with those who met criteria for a sexual desire disorder on cognitive processing of sexual cues. We assessed the cognitive processing of sexual cues in three groups: asexual individuals, heterosexual women with lifelong SIAD, and heterosexual women with acquired SIAD. Specifically, we examined group differences in visual attention to sexual images (primary endpoint) and appraisal of sexual words (secondary endpoint). All participants completed three study components: a visual attention task, Bulmer and Izuma’s sex SC-IAT [35], and sex semantic differential [35].

## Materials and methods

### Participants

A convenience sample of 42 asexual individuals (*M*_age_ = 26.67, *SD* = 5.31), nine heterosexual cisgender women with lifelong SIAD (*M*_age_ = 24.78, *SD* = 4.52), and 16 heterosexual cisgender women with acquired SIAD (*M*_age_ = 29.06, *SD* = 4.64) were recruited from the Vancouver and Toronto communities. Specifically, 10 (*M*_age_ = 27.00, *SD* = 6.99) of the 42 asexual individuals were recruited in Toronto and participated in the study at the University of Toronto Mississauga campus. Of note, although we acknowledge the diversity in experiences of sexual attraction within the ace community, given that this is the first study (as far as we are aware) to examine cognitive mechanisms underlying asexuality and sexual desire concerns, we elected for a narrow definition of asexuality. Thus, we recruited individuals who used asexual as their primary sexual identity descriptor, rather than Gray-A or demisexual. There were no notable differences in age across groups. Participants were required to meet the following inclusion criteria, which was assessed via telephone self-report: (1) 19+ years of age; (2) had normal or corrected-to-normal vision; (3) identified as asexual or heterosexual; (4) able to read and write English fluently. Participants were excluded if they reported eye diseases (e.g., macular degeneration) or color blindness. Allosexual participants were required to self-identify as heterosexual (due to the nature of the sexual stimuli used) and meet *DSM-5* diagnostic criteria for SIAD. Women who reported that their sexual concerns were present since their first sexual encounter were classified as having the lifelong subtype. In contrast, women who disclosed that their sexual difficulties began following a period of satisfactory sexual functioning were allocated to the acquired SIAD group (duration of SIAD symptoms: *M* = 4.25 years, *SD* = 3.28 years). Women with SIAD who reported genital pain were included in the analyses, as long as the pain did not fully account for their low desire. Three participants who identified as demisexual were excluded from analyses. The eye-tracking data for three asexual participants could not be used due to poor calibration (i.e., the eye-tracker was not able to recognize/track the participants’ eye movements).

### Measures

#### Phone interview

Participants were assessed for SIAD symptoms in a telephone interview conducted by a trainee completing her Master’s degree in clinical psychology. Participants were asked whether they experienced an absence/reduction in the following: sexual interest, sexual thoughts/fantasies, initiation of sexual activity or receptivity to sexual advances, responses to erotic cues (e.g., sex scene in a movie), sexual excitement/pleasure, and genital/nongenital sensations. A diagnosis of SIAD was made if women endorsed at least three of these six symptoms for a duration of six months or longer, and reported that these symptoms caused significant personal distress. Participants were also assessed for symptoms of Genito-Pelvic Pain/Penetration Disorder as part of a separate project (data not included).

#### Visual attention apparatus

Eye movements were recorded by different instruments depending on the testing site. For the Vancouver site the SensoMotoric Instruments RED 500 desktop eye-tracking system was used in conjunction with the SMI’s Experiment Suite Software program. The SMI is a remote sensor contact free eye-tracker that measures eye movements via bright and dark pupil tracking. The apparatus consists of inconspicuous external tracking hardware attached to the bottom of a stand-alone 22-inch computer monitor with a screen resolution of 1920 x 1080 pixels. The eye-tracking system works at a sampling rate of 120 Hz, has a spatial resolution of 0.03°, and a gaze position accuracy of 0.4°. The SMI automatically compensates for small head movements, so it is unnecessary to use a chin rest to immobilize the head. The apparatus is compatible with use with most eyeglasses and contact lenses.

For the Toronto site, the experiment was designed and presented in SR Research Experiment Builder software and eye movements were recorded by the SR Research Eyelink Portable Duo tracking system. The Eyelink Portable Duo can be used in either a head stabilized or remote, head free-to-move mode and measures eye movements via cornea reflection. For the current study, the head stabilized mode was employed and the participants used a chin rest for the duration of the visual attention task. Stimuli were presented on a 20-inch monitor with a resolution of 1920 x 1080 pixels. Eye movements were recorded via a second computer at 500 Hz with a spatial resolution of 0.01°, and a gaze position accuracy of 0.15°. A standard 9-point calibration and validation procedure was used, and the eye with the best spatial accuracy was selected for tracking. The apparatus is compatible for use with most eyeglasses, but not with contact lenses.

#### Implicit appraisals: Single-Category Implicit Association Task (SC-IAT)

Participants completed Bulmer and Izuma’s sex SC-IAT [35] on a laptop computer. The SC-IAT is a computer-based assessment tool that uses a classification task to measure the strength of associations held in one’s memory. IATs are widely endorsed measures of implicit associations that have consistently outperformed alternative implicit measures with regards to effect size and reliability [25]. The underlying logic of the task is that people will respond more quickly to concepts that are strongly associated in memory than those that are weakly associated. Participants classified two types of stimuli: sexual words and words representing positive or negative constructs to the corresponding superordinate category (i.e., sex, good, bad) by pressing response keys as quickly as possible. The category labels were located in the upper left- and right-hand corners of the screen for the duration of the task. The SC-IAT included two practice blocks and two experimental blocks. The experimental trials consisted of one congruent and one incongruent block of trials. For the congruent block “good” and “sex” were mapped onto the “i” key, and “bad” was mapped onto the “e” key. For the incongruent block “bad” and “sex” were mapped onto the “e” key, and “good” was mapped onto the “i” key. Practice blocks consisted of 24 trials, and experimental blocks included 72 trials, with responses divided equally over the two response keys. The word stimuli included seven generally positive (i.e., peace, glorious, joy, sunshine, smile, happy, and wonderful), seven generally negative (i.e., evil, failure, awful, horrible, terrible, agony, and nasty), and seven sexual (i.e., oral sex, penetration, erection, erotic, foreplay, climax, and fondle) words controlled for frequency and length.

#### Demographics and sexual orientation

Participants were asked to provide standard demographic information including age, education, ethnicity, romantic orientation, relationship status, and relationship length. Participants were invited to answer the question, “What is your sexual orientation?” and were provided with the following response options: asexual, bisexual, demisexual, heterosexual, lesbian/gay, pansexual, and prefer not to answer. Participants who selected *asexual* or *heterosexual* were included in all analyses. We also administered self-report measures of various sexual (i.e., sexual desire, sex-related distress, sexual aversion, genital self-image) characteristics for a separate project (data not presented).

#### Asexual identity

Participants’ scores on the Asexuality Identification Scale [41] were used to assess their asexual status. Respondents used a Likert scale to rate the applicability of various statements (e.g., I would be content if I never had sex again) to their experience, with responses ranging from 1 (*completely false*) to 5 (*completely true*). A total AIS score (12–60) was calculated by summing individual responses, with higher scores indicating a greater likelihood that the participant identifies as asexual. A cut-off score of 40/60 is typically employed to distinguish asexual from allosexual participants [41]. The AIS demonstrated excellent internal consistency in the current sample (α = 0.94).

#### Depression

The second edition of the Beck Depression Inventory (BDI-II) [42] was used to assess depressive symptoms. The BDI-II consists of 21 statements that participants rated on a 4-point Likert scale ranging from 0 to 3, and scores ≥15 are typically used to identify individuals likely to meet diagnostic criteria for depression. The BDI-II demonstrates strong convergent validity with another widely used measure of depression, the Center for Epidemiologic Studies Depression Scale (*r* = .66 –.86) [43, 44], high test-retest reliability (*r* = .73 –.96) [44], and excellent internal consistency in the current sample (α = 0.91).

#### Explicit appraisals

Attitudes towards sex were assessed using a sex semantic differential developed by Bulmer and Izuma [35]. Participants were asked to indicate where they would place “sex” between 11 antonym pairs (e.g., good, bad) by selecting one of seven unnumbered points between words. Total scores range from 11–77, with higher scores indicating stronger positive explicit appraisals of sex. The sex semantic differential demonstrated relatively good internal consistency (α = 0.85) in the current sample.

### Procedures

Of note, while we designed the overall study procedure and specified the order of experimental tasks, procedures for the sex SC-IAT were adopted from Bulmer and Izuma [35], and the forced-attention eye-tracking paradigm was adopted from Dawson and Chivers [27, 29]. Interested participants contacted the study coordinator directly via phone or email to schedule a phone screen that was conducted to assess eligibility and explain the study procedures. During the phone screen, the study coordinator inquired about sexual orientation (i.e., self-identified asexual vs. heterosexual allosexual), and conducted a brief, standardized interview to screen for major mental disorders, genital/sexual pain, and assess criteria for SIAD. Participants were assigned to one of three groups according to their answers to these questions (asexual with no sexual distress, heterosexual with lifelong SIAD, heterosexual with acquired SIAD). We consulted with an asexuality advisory group prior to data collection, to ensure our methods of assessment and procedures were acceptable to those with lived experiences of asexuality, and to refine our inclusion criteria for the asexuality sample.

Following the phone screen, the study coordinator emailed eligible participants a copy of the consent form. Individuals who were interested in proceeding with study participation contacted the study coordinator to schedule their in-lab appointment. Questionnaires, including the sex semantic differential, were completed using an online survey tool (Qualtrics) prior to in-lab experimental tasks through an individualized link sent to them via email.

Upon arrival to the laboratory located in either Vancouver or Toronto, participants were given an overview of study procedures by the coordinator and provided written consent. Participants were seated in front of a laptop and were asked to complete a brief cognitive task. The study coordinator started the computer program that presented the SC-IAT, asked participants to communicate via intercom if they had questions or concerns, and left the room to allow the participants privacy. Instructions appeared on the screen, informing participants to press the “e” key (left response key) or the “i” key (right response key) to categorize words into groups (good, bad, sex) as quickly as possible without making errors. The first block (practice) familiarized participants with the task and asked them to classify words using the response keys, with “sex” and “good” mapped onto the “i” key, and “bad” mapped onto the “e” key. The second (experimental) block had the same format. The third (practice) block’s instructions informed participants that the labels changed sides, with “good” mapped onto the “i” key, and “bad” and “sex” mapped onto the “e” key, but that the task had not changed. The fourth (experimental) block had the same format. After participants completed the last block, a message appeared on the screen telling them that they had finished the task.

We elected to administer the SC-IAT prior to the visual attention task given 1) data suggesting that performance on implicit appraisal paradigms is affected by contextual information [45–47], 2) studies indicating that some asexual participants experience disgust in response to sex [12, 38, 48], and 3) our previous experience testing asexual participants who covered their eyes when presented with sexual imagery. Thus, we were concerned that if participants completed the visual attention task prior to the SC-IAT, then groups’ performance on the latter task would be affected by their emotional responses to the erotic images, with these stimuli being more likely to elicit disgust for the asexual group. Of note, for all participants there was an approximately 10-minute delay between completion of the SC-IAT and the beginning of the eye-tracking task, during which the study coordinator completed the preparatory steps outlined hereafter.

The study coordinator entered the room with permission from the participant via intercom. She removed the laptop and the eye-tracker system was set-up and calibrated in the private room in preparation for the visual attention task. Calibration of the device entailed having participants follow a circle that moved around the video display screen with their eyes. After set-up was complete and a verbal explanation of the task was provided, participants were left alone in the private testing room. The study coordinator communicated with them through an intercom system connected to the adjacent room for the duration of the task.

**Fig 2:**
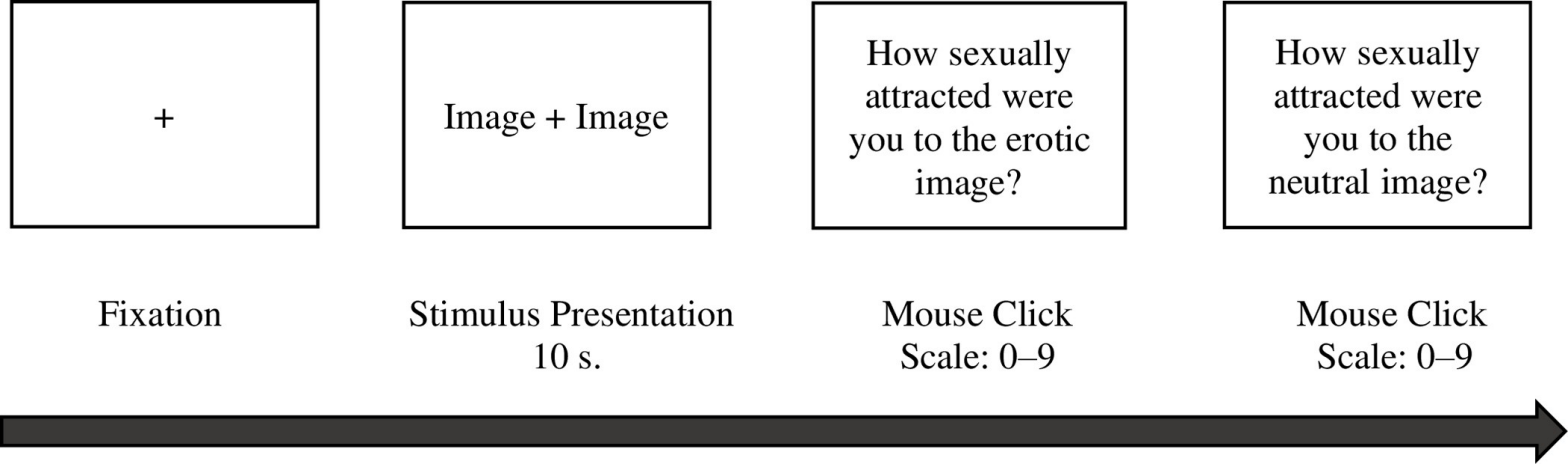
Sample experimental trial in the eye-tracking task. Depiction of the time sequence and visual presentation of a single trial.

The forced-attention paradigm presented 20 experimental trials. Each experimental trial presented a pair of images, including one explicit erotic and one non-erotic, neutral image. Erotic images depicted a nude male actor and a nude female actor engaging in a sexual act, whereas non-erotic images showed a clothed man and woman engaging in a non-sexual, non-romantic activity. Each stimulus pair were presented side-by-side on the computer monitor (e.g., erotic image: middle-left; non-erotic image: middle-right), equidistant from the center of the screen. The stimuli were matched for size, brightness, contrast, and color to limit biased patterns of attention induced by low-level image features [27, 29]. Participants were instructed to view the images as they would naturally in situations outside the lab. For each stimulus display, a fixation cross appeared on the screen and was followed by the presentation of the image pair for 10 seconds (see Fig 2 for a trial overview). After viewing each pair of images, participants completed a picture rating task and used a computer mouse located on a table beside the testing chair to rate their sexual attraction to each erotic and non-erotic image on a 10-point scale ranging from 0 (*not at all sexually attracted*) to 9 (*very sexually attracted*). Of note, during study debriefing several asexual participants reported that they were unclear about the type of sexual attraction this task referred to, while none of the women with SIAD raised these concerns. As a result of the questionable validity of this measure, we elected to not include those results here.

During an early data presentation, the possibility was raised that participants may not fixate on an image but instead attend to it covertly “out of the corner of their eye”. As a check on covert attention in the absence of subsequent overt attention (i.e., fixation to an object), we added a memory task to check whether participants’ memory for certain images corresponded to their controlled attention to said images. Evidence of covert attention would be apparent if a participant did not look at sexual images in the eye-tracking task, but performed similarly to others who looked at those images, when sorting sexual images in the image classification task.

Following the eye-tracking task the study coordinator re-entered the testing room, asked participants to be seated in front of a laptop computer away from the eye-tracking equipment, and informed them that they would be completing the memory task. The study coordinator started the computer program that presented this task and left the room to allow the participants privacy. Instructions appeared on the screen telling participants to press the “z” key if they recognized the image presented from the eye-tracking task (i.e., it is an old image) and to press the “/” key if they did not recognize the image (i.e., it is a new image). Participants viewed and classified 80 images (40 erotic, 40 neutral). Half of the images were selected from the eye-tracking task, and the remaining 40 were not included in this task but had a similar visual style (i.e., erotic images depicted a nude male actor and a nude female actor engaging in sexual activity, and non-erotic images showed a man and woman in casual clothes engaging in a non-sexual, non-romantic activity).

When the testing session was finished (1hr), participants were provided with a debriefing form and a verbal explanation of the purpose of the study, and given a remuneration of $25. This study was approved by the Behavioral Research Ethics Board at the University of British Columbia, #H18-03236, the Vancouver Coastal Health Hospital Research Ethics Board, and the Institutional Ethics Board at the University of Toronto, #38252. The study was pre-registered and is available online at the Open Science Framework (doi:10.17605/OSF.IO/ZYS86).

### Data reduction and analysis

#### Power calculations

The primary aim of this study was to evaluate group differences in controlled visual attention to sexual stimuli between asexual individuals and women with lifelong and acquired SIAD. Previous studies employing forced-attention paradigms of a similar nature [27, 29] found large differences between predominantly/exclusively androphilic (i.e., attracted to men) and gynephilic (i.e., attracted to women) groups’ controlled visual attention to preferred and nonpreferred sexual cues. Given these results, we predicted effects that were large in magnitude. A priori power analyses for one-way Analyses of Variance (ANOVAs) examining group differences in dwell time and number of fixations to sexual cues (i.e., sexual images and areas of sexual contact) revealed that to achieve the predicted large effect size (*η*^2^ = 0.14) with power set at .80, an *N* of 66 (*n* = 22 per group) would be needed to observe differences. Data collection was abruptly halted due to COVID-19, resulting in a smaller sample size than anticipated. Given that our sample size for the SIAD groups did not reach our goal, we interpreted null effects with caution. All power analyses were conducted using G*Power 3 software [49].

We focused on effect sizes rather than null hypothesis significance testing and *p* < .05 [50], and have reported eta-squared and Cohen’s *d* for all group comparisons presented in this manuscript. Inferential tests and *p*-values were included for the readers’ consideration.

#### Data reduction

The stimulus display (i.e., screen) for each trial was coded based on three regions of interest: 1) erotic image; 2) non-erotic image; 3) area of sexual contact (i.e., penile-vaginal penetration, cunnilingus, or fellatio). Dwell time represents the total amount of time spent (in seconds) in the regions of interest across the total stimulus presentation time, and number of fixations represents the number of times a participant’s gaze landed in a region of interest. To examine our first set of hypotheses related to visual attention, four dependent variables were calculated: proportion of dwell time to 1) erotic image vs. non-erotic image, 2) area of sexual contact vs. screen; proportion of fixations to 3) erotic image vs. non-erotic image, and 4) area of sexual contact vs. screen. For each trial, every participant had a score for each of the four aforementioned proportion variables. These scores were averaged across the 20 experimental trials to result in mean scores for each variable. Longer dwell times and greater number of fixations towards a target are indicative of a controlled attention bias for the target.

SC***-***IAT effects were operationalized by D-scores, which range from -2, representing a strong negative association with the construct of interest, and +2, indicative of a strong positive association. D-scores were calculated in INQUISIT using Greenwald and associates’ IAT scoring algorithm [51]; specifically, data from practice and experimental trials were included, while trials with latencies >10,000 ms were excluded. In accordance with Greenwald et al. [51], error trials were handled by allowing participants to correct their incorrect responses. Each participant’s mean latency was divided by their pooled SD for all trials in order to account for variability in response latencies. Finally, D-scores were calculated by subtracting the mean latencies of the congruent blocks (i.e., bad and sex were paired) from the incongruent blocks (good and sex were paired). Thus, for this study, positive D-scores indicated stronger positive associations with sexual stimuli, whereas negative D-scores suggested more negative associations. To operationalize explicit appraisals of sex, we averaged participants’ ratings on the 11 questions of the sex semantic differential, and higher scores on this measure represented more positive attitudes towards sex.

#### Data analysis

See Table 1 for hypotheses and corresponding data analyses.

**Table 1:** Hypotheses and data analyses for primary and secondary endpoints.

Hypotheses	Dependent Variable
**1: Visual Attention**	
Asexual individuals will non-preferentially view sexual and non-sexual stimuli whereas women with lifelong and acquired SIAD will have longer dwell times and more fixations to sexual relative to neutral images	1. Proportion of dwell time to erotic vs. non-erotic image^a^
	2. Proportion of fixations to erotic vs. non-erotic image^b^
Asexual individuals will exhibit shorter dwell times and make fewer fixations to the area of sexual contact relative to women with lifelong and acquired SIAD	1. Proportion of dwell time to sexual contact vs. screen^a^
	2. Proportion of fixations to sexual contact vs. screen^b^
**2: Implicit Appraisals**	
Asexual individuals and women with lifelong SIAD will exhibit less positive implicit appraisals of sexual stimuli than women with acquired SIAD	SC-IAT D-score
**3: Explicit Appraisals**	
Asexual individuals will display less positive explicit appraisals of sex than women with lifelong or acquired SIAD	Sex semantic differential scores

One-way ANOVAs were conducted to test all hypotheses, with group (asexual, lifelong SIAD, acquired SIAD) as the independent variable. Main effects for one-way ANOVAs were followed up with Tukey’s HSD post-hoc comparisons.

^a^ Violations of homogeneity of variance were detected for one-way ANOVAs, *Welch’s F* and Games-Howell post-hoc comparisons were reported.

^b^ In order to avoid redundancy, we did not report findings for total number of fixations as they were consistent with results for total dwell time.

## Results

### Sample characteristics

Asexual individuals (*n* = 42), women with lifelong SIAD (*n* = 9), and women with acquired SIAD (*n* = 16) were similar on several demographic characteristics, including age, relationship length, education, and ethnicity (Table 2). A one-way ANOVA revealed that asexual individuals had higher scores on the AIS relative to women with lifelong and acquired SIAD, *F*(2,64) = 62.84, *p* < .001, *η*^2^ = 0.66. Regarding gender, five asexual participants identified as non-binary and disclosed trans experience (i.e., their gender identity did not align with their sex at birth), whereas all participants in the SIAD groups identified as cisgender women. A greater number of asexual participants reported being single (73.2%) relative to women with lifelong SIAD (11.1%) and women with acquired SIAD (12.5%), χ^2^ = 23.25, *p* < .001. Of note, 24.4% of the asexual sample identified as aromantic (i.e., do not experience romantic attraction to others) relative to none of the women with SIAD, who all described themselves as romantic, χ^2^ = 7.19, *p* = .028. The majority of participants were white, with no significant group differences in the distribution of individuals across ethnicity categories. Although, one person in the asexual group self-identified as Southeast Asian, with no women in the SIAD groups identifying in this way. Similarly, one woman with lifelong SIAD self-identified as Hispanic, and one woman with the acquired subtype self-identified as First Nations. Considering educational attainment, the majority of the sample had either attended some college (28.4%) or completed a college or post-graduate degree (68.7%). Regarding socioeconomic status, the median annual income across groups was $40,000 - $59,999. Notably, more women in the acquired SIAD group reported an annual income >$60,000 (80%) relative to asexual individuals (36.4%) and women with lifelong SIAD (37.5%), χ^2^ = 6.12, *p* = .047.

**Table 2:** Sociodemographic information for participants.

	Asexual	Lifelong SIAD	Acquired SIAD
Age *M (SD)*	26.7 (5.3)	24.8 (4.5)	29.1 (4.6)
Gender identity (%)			
Woman	87.2	100	100
Non-binary	12.8	0	0
Trans-experience (%)	13.2	0	0
Romantic Orientation (%)*			
Aromantic	24.4	0	0
Romantic	75.6	100	100
Relationship status (%)*			
Single	73.2	11.1	12.5
Dating/committed relationship	26.8	88.9	87.5
Relationship length in yrs. *M* (*SD*)	4.7 (5.2)	3.4 (2.3)	5.3 (3.0)
AIS *M* (*SD*)*	48.4 (7.0)	30.1 (8.0)	25.8 (8.4)
Sexual assault history (%)	52.5	44.4	40.0
BDI-II *M* (*SD*)	11.0 (10.9)	14.2 (12.9)	12.4 (8.9)
Ethnicity (%)			
East Asian	19.0	22.2	31.3
South Asian	4.8	11.1	6.3
Southeast Asian	2.4	0	0
First Nation	0	0	6.3
Hispanic	2.4	11.1	6.3
Arab/West Indian	0	11.1	0
White/Caucasian	59.5	44.4	43.8
Other	11.9	0	6.3
Level of education (%)			
High school	4.8	0	0
Attended some college	33.3	33.3	12.5
College degree	50.0	44.4	56.3
Post-graduate degree	11.9	22.2	31.3
Income category (annual)*			
<$20,000	21.2	37.5	13.3
$20,000 –$39,999	18.2	12.5	0
$40,000 –$59,999	24.2	12.5	6.7
$60,000 –$79,999	15.2	12.5	0
$80,000 –$99,999	6.1	0	33.3
$100,000 –$119,999	6.1	12.5	13.3
$120,000 –$139,999	3.0	0	0
$140,000 –$159,999	6.1	12.5	20.0
>$160,000	0	0	13.3

Abbreviation: AIS = Asexuality Identification Scale; BDI-II = Beck Depression Inventory. Table presents comparisons between asexual individuals (*n* = 42), women with lifelong SIAD (*n* = 9), and women with acquired SIAD (*n* = 16). One-way ANOVAs evaluated group differences in age, relationship length, AIS, and BDI-II scores. Chi-square tests of homogeneity examined group differences in gender-identity, trans experience, romantic orientation, relationship status (single vs. dating/partnered), sexual assault history, ethnicity (White/Caucasian vs. all other categories), level of education (no college degree vs. college degree), and annual income (above vs. below median income–$40,000 –$59,999).

*Groups differed on variable of interest, *p* < .05.

### Hypothesis 1: Visual attention

#### Erotic vs. non-erotic image

For the mean proportion of dwell time to the erotic image, the one-way ANOVA revealed a main effect of group, and a Welch correction was used to account for a homogeneity of variance violation, *Welch’s F*(2, 21.40) = 18.17, *p* < .001, *η*^2^ = 0.31. Games-Howell post-hoc tests revealed that asexual individuals had shorter dwell times to the erotic image relative to women with lifelong SIAD (*p* = .005, *d* = 1.30) and acquired SIAD (*p* < .001, *d* = 1.54). Women with different SIAD subtypes exhibited similar dwell times to the erotic image (*p* = .997, *d* = 0.00).

#### Area of sexual contact vs. rest of screen

Of note, to ensure that eye-tracking systems used at different sites did not affect our visual attention findings, we examined the effect of site on these endpoints and found no differences between asexual participants recruited in Toronto vs. Vancouver on the proportion of dwell time to the erotic image, *t*(37) = -1.07, *p* = .290, or the area of sexual contact, *t*(37) = -1.44, *p* = .159.

**Fig 3:**
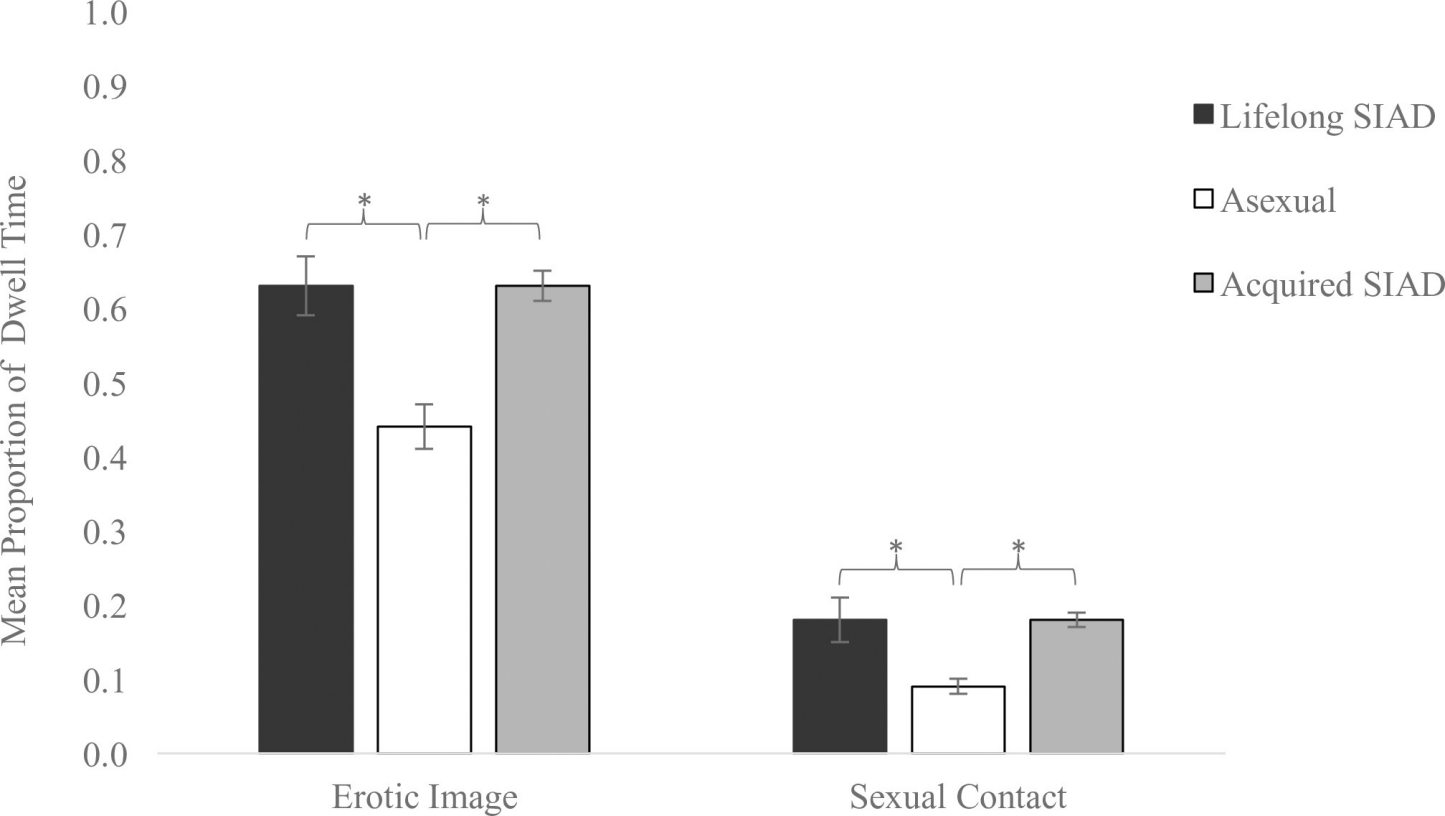
Proportion of dwell time to the erotic image and area of sexual contact. Asexual individuals (*n* = 39) had shorter dwell times (s) to the erotic image and area of sexual contact relative to women with lifelong SIAD (*n* = 9) and women with acquired SIAD (*n* = 16). Error bars represent standard error of the mean. *p < .05.

For the proportion of dwell time to the area of sexual contact, a one-way ANOVA revealed a main effect of group, *F*(2,61) = 18.50, *p* < .001, *η*^2^ = 0.38. Tukey HSD post-hoc tests reflected that asexual individuals had shorter dwell times to the area of sexual contact relative to women with lifelong SIAD (*p* < .001, *d* = 1.24) and acquired SIAD (*p* < .001, *d* = 1.80). Women with lifelong and acquired SIAD spent a similar proportion of time attending to the area of sexual contact (*p* = .975, *d* = 0.00). See Fig 3 for mean proportions of dwell time to the erotic image and area of sexual contact for all groups.

#### Supplementary analysis: Memory task

In order to ensure that group differences in visual attention to sexual cues were not accounted for by asexual individuals employing covert attention (i.e., looking out of the corner of one’s eye without fixating) to sexual images, we conducted an independent samples t-test to examine group differences in performance on the memory task between asexual individuals and women with SIAD, using the proportion of sexual images classified correctly as the dependent variable. Thirty-six participants (20 asexual, 16 with SIAD) completed this task, as it was added to the study after data collection was underway. Given similarities between women with lifelong and acquired SIAD on controlled visual attention endpoints, we collapsed these groups for this comparison. The analysis revealed that women with SIAD classified sexual images more accurately (*M* = 0.90, *SD* = 0.08) than asexual participants (*M* = 0.78, *SD* = 0.11), *t*(34) = -3.93, *p* < .001, *d* = 1.24.

### Hypothesis 2: Implicit appraisals

The one-way ANOVA did not reveal a main effect of group, *F*(2,64) = 1.34, *p* = .270, *η*^2^ = 0.04. Tukey HSD follow-up comparisons indicated that D-scores were comparable between asexual individuals (*M* = -0.17, *SD* = 0.38), women with lifelong SIAD (*M* = -0.21, *SD* = 0.29), and women with acquired SIAD (*M* = 0.00, *SD* = 0.37; *p*s = .301 –.943, *d*s = 0.12–0.63).

### Hypothesis 3: Explicit appraisals

**Fig 4:**
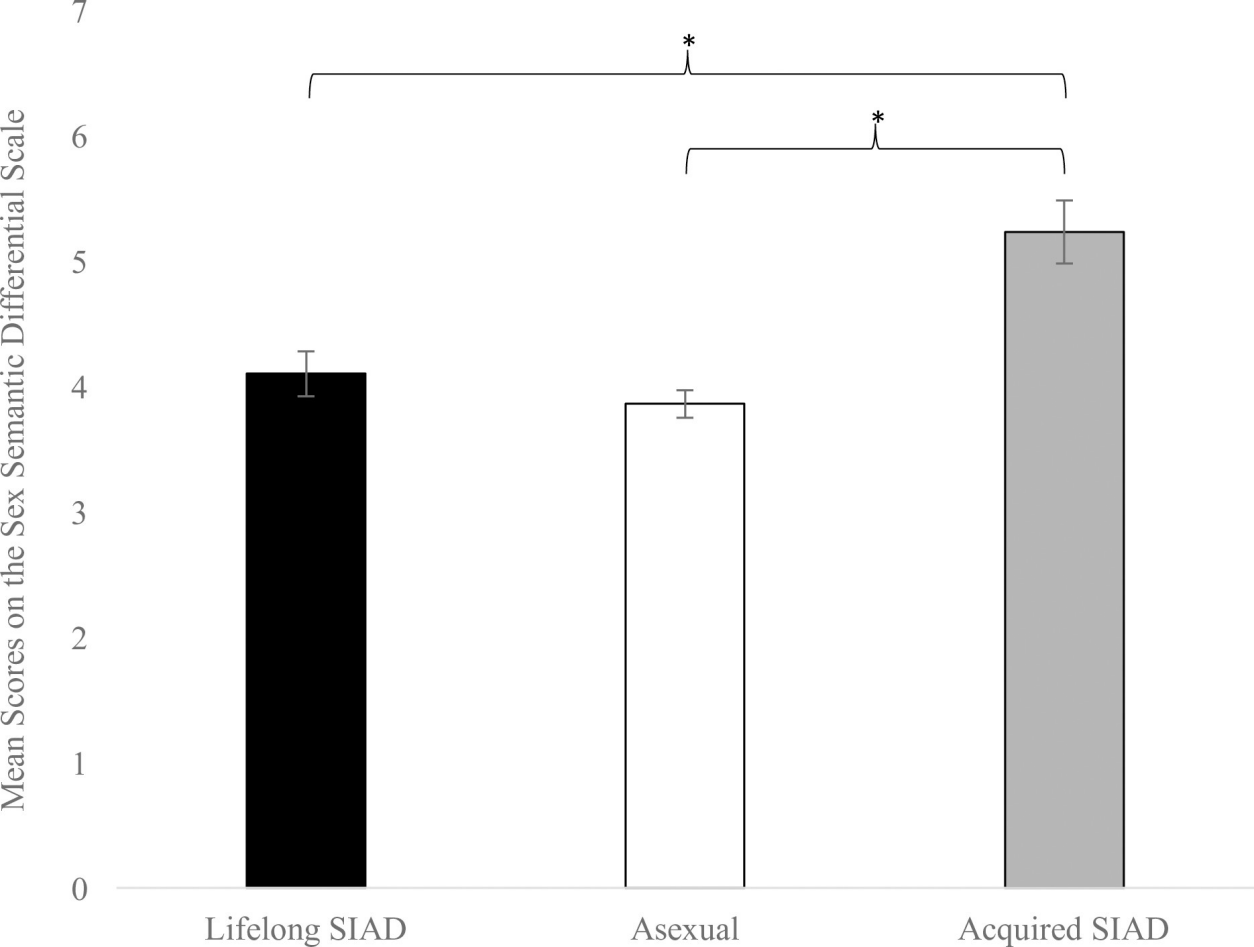
Sex semantic differential scores. Asexual individuals (*n* = 42) and women with lifelong SIAD (*n* = 9) had lower average sex semantic differential scores compared to women with acquired SIAD (*n* = 16). Error bars represent standard error of the mean. *p < .05.

For scores on the sex semantic differential, a one-way ANOVA revealed a main effect of group, *F*(2,62) = 18.12, *p* < .001, *η*^2^ = 0.37. As seen in Fig 4, Tukey HSD follow-up comparisons reflected that women with acquired SIAD had higher average scores on the sex semantic differential relative to asexual individuals (*p* < .001, *d* = 1.59) and women with lifelong SIAD (*p* = .002, *d* = 1.43). There were minimal differences between the average sex semantic differential scores for the asexual and lifelong SIAD groups (*p* = .680, *d* = 0.38).

## Discussion

### Summary of findings

This study is the first, as far as we are aware, to explore group differences in the cognitive processing of sexual cues between asexual individuals, heterosexual women with lifelong SIAD, and heterosexual women with acquired SIAD by evaluating their visual attention to and appraisals of sexual stimuli. Regarding visual attention, we found a main effect of group consistent with our hypotheses. Women with both SIAD subtypes exhibited a preference for erotic images, such that they demonstrated longer dwell times to erotic relative to non-erotic images, whereas asexual individuals did not exhibit this preference. Further, women with lifelong and acquired SIAD committed their attention to areas of sexual contact within erotic images to a greater degree than asexual individuals, who had shorter dwell times toward this region of interest compared to SIAD groups. In line with our hypotheses, we did not detect differences in gaze behavior towards sexual cues (i.e., sexual images or areas of sexual contact) between women with different SIAD subtypes. For implicit appraisals of erotic cues, the findings did not support our hypotheses in that there were no notable group differences in performance on the sex SC-IAT. For explicit appraisals of sex, our hypotheses were partially supported in that we found the predicted difference between the acquired SIAD and asexual groups with the former showing higher average sex semantic differential scores, indicative of more positive attitudes towards sex. However, contrary to our predictions, we found no differences between the explicit appraisals of sex between asexual individuals and women with lifelong SIAD.

### Asexuals do not show visual attention preference for erotic images

Asexual individuals did not exhibit a controlled attention bias for erotic cues whereas women with lifelong and acquired SIAD demonstrated a sex-specific preference. These findings are consistent with research suggesting that controlled visual attention to sexual cues is influenced by the direction of one’s sexual attractions [27, 29, 52–54]. Given that asexual persons do not experience sexual attraction to others, it is likely that images depicting sexual activity were not as motivationally salient to this group, which may have resulted in asexual individuals allocating less controlled attention to these cues.

When interpreting these findings, it is important to consider the variability in asexual individuals’ self-described attitudes towards sex, ranging from disinterest to disgust [11, 12, 38]. This diversity in evaluations of sex may have been reflected in the sizeable range of dwell times to erotic images and areas of sexual contact. It is possible that asexual individuals who were disinterested in but not repulsed by sexual activity distributed their attention relatively evenly between the image types, whereas asexual participants who were sex-repulsed may have actively avoided the erotic images. Future studies should investigate whether attention to erotic cues is affected by evaluations of sex.

There were negligible differences in gaze behavior to sexual cues between women with different SIAD subtypes. It is likely that these groups’ heterosexual orientation accounted for their similar patterns of controlled visual attention to sexual stimuli, given that all erotic images depicted men and women engaging in various sexual acts, the self-reported desired interactions for these groups. Alternatively, sex may be an activity that women with SIAD participate in to strengthen their romantic partnerships in spite of their low desire, and studies have highlighted emotional motives for sex for women with SIAD [55]. Therefore, it may be the case that women with SIAD attended to the erotic images due to their emotional, rather than purely sexual, qualities. However, the lack of differences in gaze behavior between SIAD subtypes should be interpreted with caution, given the small number of women with lifelong SIAD (*n* = 9) and consequent power concerns.

### Implicit appraisals

Asexual individuals and women with lifelong SIAD did not exhibit markedly less positive implicit appraisals of sexual cues relative to women with acquired SIAD. However, it should be noted that both asexual individuals and women with lifelong SIAD displayed slightly negative implicit appraisals of erotic cues, whereas women with acquired SIAD demonstrated relatively neutral appraisals of these stimuli. It is possible that we did not detect the predicted group differences due to low power, and our results are inconsistent with those of Bulmer and Izuma [35], who used the same SC-IAT and found that asexual participants showed more negative implicit appraisals of sexual word stimuli than their allosexual counterparts, and this difference was large in magnitude. Further, our findings stand in contrast to other research studies whose IAT results have distinguished participants from different sexual orientation groups [33, 34].

Focusing on women with SIAD, it is unsurprising that these groups displayed negative–neutral implicit appraisals of sexual words, given their sexual difficulties. Women in both SIAD groups disclosed that their sexual concerns were a source of significant distress in their lives. Thus, the sexual words may have triggered negative affective reactions for these women. Notably, a recent study using a similar implicit appraisal paradigm found that automatic associations with sexual images were not correlated with nor predictive of women’s self-reported levels of sexual desire [56]. Therefore, an alternate possibility is that implicit appraisals of erotic cues are not meaningfully affected by levels of desire.

### Explicit appraisals

Asexual individuals held less positive attitudes towards sex than women with acquired SIAD. Notably, women with lifelong SIAD also held less positive evaluations of sex than women with acquired SIAD, and their scores on the sex semantic differential were similar to those of asexual individuals. For the asexual sample, our findings suggest that asexual individuals hold somewhat negative or neutral impressions of sex and were comfortable reporting these attitudes in a survey, replicating Bulmer and Izuma’s findings [35]. This result is in line with the asexual community’s rejection of compulsory sexuality, the widespread assumption in Western society that all people are sexual and that sexual relationships are of great importance [5]. However, there was substantial variability in asexual individuals’ mean sex semantic differential scores, reflecting the diversity in opinions about sex seen in asexual communities [11, 12, 38].

While we hypothesized that women with lifelong and acquired SIAD would report similarly positive explicit appraisals of sex, our results revealed that women with lifelong SIAD reported attitudes that were neither positive or negative, with their sex semantic differential scores falling near the midpoint of the scale. In contrast, women with acquired SIAD were sex-positive, and their mean sex semantic differential scores were similar to those of heterosexual men and women without sexual concerns [35]. Thus, it is possible that this measure is tapping into holistic attitudes towards sex developed over the lifespan, which may be shaped by the number and tone of past sexual experiences. However, the variability in sex semantic differential scores for the acquired SIAD group was similar to that of the asexual sample, indicating that while many reported positive attitudes towards sex, this was not the case for all women.

### Implications for classification

When considering the implications of our findings for the classification of asexuality and SIAD, we have elected not to focus on group similarities due to low power concerns. Instead, we will focus on the demonstrated group differences. Our findings revealed that asexual individuals did not gaze preferentially at erotic images, whereas women with SIAD did. Given research demonstrating that controlled attention to an erotic stimulus is indicative of sexual attraction to that cue [27, 29], our data provide preliminary support for asexual persons’ lack of sexual attraction to others. Second, for explicit appraisals, our findings suggest that while women with acquired SIAD hold positive attitudes towards sex, asexual individuals and women with lifelong SIAD likely have more negative or neutral feelings towards sexual activity. However, because our study is the first to compare the cognitive processing of sexual cues between asexual and SIAD groups, our results require replication.

### Clinical implications

We have three clinical recommendations. First, when conducting an intake interview, clinicians treating low desire could use AIS [41] questions to structure a discussion about the client’s experience of sexual attraction to help them ascertain whether an ace identity fits their experience. However, we caution against drawing conclusions about the client’s ace status from their AIS total score, as a recent analysis of the 2017 Ace Community Survey data revealed that while approximately 91% of asexual-identifying respondents scored ≥40, this recommended cut-off captured only 67% of Gray-As [57]. Second, clinicians should carefully ascertain the source of clients’ sex-related distress. Specifically, clinicians should query to discover whether the client’s distress emerges from their desire to be more sexual, or is a response to a perceived need to meet partner or societal expectations of sexuality. Third, if clients are unfamiliar with asexuality, clinicians should direct them to AVEN and could also connect them with local ace meetup groups if they are interested in further exploring this identity. Our study strengthens the need to differentiate asexuality and lifelong SIAD in the clinical setting, since failure to do so may result in unnecessary and possibly harmful treatment recommendations made to asexual individuals.

### Future directions

A major limitation of this research is the small sample size, particularly with respect to heterosexual women with lifelong SIAD (*n* = 9). Thus, minimal differences between women with lifelong SIAD and other groups must be interpreted with caution given limited power. However, it should be noted that women with this variant of SIAD are often not included in research studies given the low prevalence of this presentation of low desire. To our knowledge, other than our study only Brotto and colleagues’ investigation [19] differentiated women with acquired and lifelong SIAD, with the latter group making up only 28% of the sample. Future studies should aim to identify characteristics that distinguish women with lifelong SIAD from those with acquired SIAD to better identify their treatment needs.

In terms of our eye-tracking outcomes, we did not account for implied social presence (e.g., changes in behavior due to the knowledge that one’s eye movements were being recorded) or participants’ knowledge about the study’s purpose. Although the effects of implied social presence on visual attention is a nascent research area, recent studies have revealed that knowing one is being “watched” may influence gaze behavior to erotic stimuli [58, 59]. Thus, future studies may wish to investigate how implied social presence and awareness of the research question affects gaze behavior towards sexual cues for asexual persons as well as women with sexual difficulties. Considering other factors that may have affected our eye-tracking findings, it is possible that groups preferred (or avoided) images of certain sexual acts and not others. However, given that our 20 erotic images included 10 depicting penile-vaginal intercourse, but only five showing fellatio and cunnilingus, we did not have a sufficient number of images to investigate controlled attention preferences for certain types of sexual activity, and this presents an interesting question for future studies.

We did not examine the effect of participants’ romantic orientation on their cognitive processing of sexual cues, given that the majority of our sample identified as romantic, with only *n* = 10 asexual participants reporting aromanticism. To our knowledge, the effect of romantic attraction (or lack thereof) on the cognitive processing of sexual cues has yet to be studied. Notably, a recent study revealed that romantic asexual individuals differ from their aromantic counterparts on several sex-related variables [15]. Specifically, romantic asexual persons were more likely to have been in a relationship, had a greater number of sexual and romantic partners, reported more partner-oriented desire, and more frequent kissing than aromantic asexual individuals [15]. Considering these findings, future studies should examine whether asexual persons’ romantic orientation affects their cognitive processing of sexual stimuli.

We did not recruit individuals who primarily identified as Gray-A, and excluded participants who identified as demisexual. As a result, these findings cannot be generalized to and are not representative of all people within ace communities. Researchers should examine whether groups encompassed by the ace umbrella (i.e., asexual, Gray-A, demisexual) differ in their cognitive processing of sexual cues to better comprehend if and how people located at various positions on the ace spectrum are dissimilar.

Lastly, participants’ sex, frequency of sexual activity and the quality of past sexual experiences were not assessed in the current study, as the aim of the project was to investigate group differences in cognitive processing of sexual cues. However, these variables may influence visual attention to and appraisals of erotic stimuli. Researchers should explore whether participants’ reported sex, the quantity of sexual activity, or the positive/negative outcomes of one’s sexual experiences predict cognitive processing of sexual words and imagery, especially in clinical samples.

## Conclusions

The present findings provide new data showing that heterosexual women with lifelong and acquired SIAD display a controlled visual attention preference for sexual cues, whereas asexual persons do not. Moreover, this study sheds light on asexual individuals’ and women with SIAD’s appraisals of sexual stimuli, and revealed that while all three groups displayed negative–neutral implicit associations with sexual words, women with acquired SIAD reported more positive attitudes towards sex relative to other groups. These findings reveal that disentangling asexuality and lifelong SIAD may prove challenging for clinicians. Thus, practitioners should carefully consider whether clients presenting with longstanding sexual disinterest are on the ace spectrum or meet diagnostic criteria for lifelong SIAD, as the former group would likely not benefit from treatment. Future studies should aim to better understand how the experiences of women with lifelong SIAD differ from their counterparts with the acquired subtype in an attempt to develop treatments that better meet their needs.
